# Long-term clinical efficacy of Marshall vein ethanol infusion combined with catheter ablation for persistent AF

**DOI:** 10.3389/fcvm.2026.1809308

**Published:** 2026-05-12

**Authors:** Wei-Li Ge, Yuan Zhang, Yang-Ming Mao, Yi-Fei Lu, Tao-Hsin Tung, Su-Hua Yan

**Affiliations:** 1 Department of Cardiology, Taizhou Hospital of Zhejiang Province Affiliated to Wenzhou Medical University, Zhejiang, China; 2 Evidence-Based Medicine Center, Taizhou Hospital of Zhejiang Province, Wenzhou Medical University, Linhai, China; 3 Department of Cardiology, Shandong Provincial Qianfoshan Hospital, Shandong University, Shandong, China; 4 Shandong Medicine and Health Key Laboratory of Cardiac Electrophysiology and Arrhythmia, Department of Cardiology, The First Affiliated Hospital of Shandong First Medical University, Jinan, China

**Keywords:** catheter ablation, ethanol infusion, long-term outcomes, persistent atrial fibrillation, vein of marshall

## Abstract

**Background:**

Recurrent atrial arrhythmia after radiofrequency catheter ablation (RFCA) for persistent atrial fibrillation (AF) necessitates better strategies. Ethanol infusion of the vein of Marshall (EI-VOM) is an emerging adjunct, but long-term efficacy data combined with RFCA is limited. This study compared long-term arrhythmia-free survival between RFCA combined with EI-VOM vs. RFCA alone in persistent AF patients and analyzed outcome predictors.

**Methods:**

Persistent AF patients were divided into EI-VOM combined with RFCA (EI-VOM + RFCA, *n* = 71) and RFCA alone (RFCA, *n* = 41) groups. Both groups underwent bilateral pulmonary vein isolation and linear ablation. The EI-VOM + RFCA group received ethanol infusion into the vein of Marshall before standard RFCA.

**Results:**

Freedom from atrial arrhythmia recurrence rates in the EI-VOM + RFCA group were significantly higher at all time points: 87.32% at 12 months, 80.25% at 24 months, and 77.46% at 36 months post-procedure. Corresponding rates in the RFCA alone group were lower: 78.05%, 65.85%, and 58.54%. Multivariate analysis identified receiving EI-VOM therapy and a smaller left atrial diameter as independent predictors of maintaining sinus rhythm long-term.

**Conclusion:**

Combining EI-VOM with standard RFCA significantly reduces atrial arrhythmia recurrence compared to RFCA alone in patients with persistent AF, demonstrating superior long-term effectiveness. EI-VOM is an independent predictor of successful sinus rhythm maintenance.

## 1 Introduction

Atrial fibrillation (AF), the most prevalent form of sustained cardiac arrhythmia, poses significant clinical management challenges, particularly persistent AF. Catheter ablation (CA), particularly pulmonary vein isolation (PVI), has emerged as a cornerstone therapy for AF, with high success rates in paroxysmal AF (PAF) (1). However, its efficacy diminishes in patients with persistent AF (PeAF), with recurrence rates exceeding 30% within one year post-ablation (2, 3). This discrepancy stems from the complex pathophysiology of PeAF, which is characterized by extensive atrial remodeling, nonpulmonary vein triggers, and epicardial conduction gaps that evade endocardial ablation (4).

To address these limitations, adjunctive strategies such as linear ablation [e.g., mitral isthmus (MI) and roof lines] have been integrated into PVI. Subsequently, upgraded strategies have been developed to enhance the efficacy of ablation for persistent AF, including bilateral circumferential pulmonary vein antral isolation (2C) and three linear ablation sets (3L) (4). However, the combination of PVI and linear ablation has not shown superiority over PVI alone in rhythm control, primarily because of difficulties in creating durable lesions, particularly in the MI. Nevertheless, a durable MI block remains elusive owing to anatomical complexities, including thick myocardial tissue and overlapping epicardial structures, such as the vein of Marshall (VOM) and coronary sinus musculature (5). The VOM, an embryological remnant harboring sympathetic nerve fibers and arrhythmogenic substrates, has garnered attention as a therapeutic target. Ethanol infusion into the VOM (EI-VOM) induces localized chemical ablation, disrupts autonomic innervation, and facilitates transmural lesion formation, thereby enhancing MI block rates and reducing AF recurrence (6–10).

Recent trials, including the VENUS-AF and PROMPT-AF trials, have demonstrated the short-term benefits of EI-VOM combined with PVI (11, 12). However, long-term outcomes beyond 12 months remain understudied, and the interplay between EI-VOM and left atrial remodeling is poorly characterized. This study compared the 3-year arrhythmia-free survival of EI-VOM combined with radiofrequency catheter ablation (RFCA) vs. RFCA alone in patients with PeAF, while identifying predictors of recurrence, such as left atrial diameter.

## 2 Methods

### 2.1 Study characteristics

**Figure 1:**
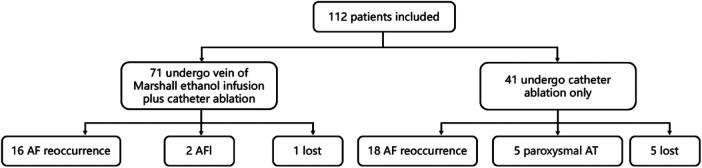
Flow diagram of patient enrollment and procedure.

This single-center retrospective study compared the effectiveness of rhythm control with two ablation strategies for patients with persistent AF: RFCA alone or with EI-VOM (EI-VOM + RFCA). The RFCA group utilizes a fixed anatomical ablation strategy, including pulmonary vein isolation, left atrial roof ablation, MI ablation, and tricuspid isthmus ablation. In the EI-VOM RFCA group, an additional EI-VOM procedure was performed along with the RFCA strategy; Based on the available sample quantity, the flow chart of the trial is presented in Figure 1. This was a retrospective study, and no *a priori* sample size calculation was performed. All eligible patients meeting the inclusion criteria during the study period were included.

This cohort study included patients who underwent catheter ablation at Taizhou Hospital of Zhejiang Province between December 2019 and December 2021. All participants provided informed consent, and a minimum follow-up of 36 months was conducted after discharge. Patients aged 18–80 years were included in the study. Patients with structural cardiac abnormalities detected via transthoracic echocardiography, including rheumatic heart disease, mechanical or bioprosthetic valve replacement, hypertrophic cardiomyopathy, mitral valve repair, dilated cardiomyopathy, left ventricular dilation (≥60 mm), or left atrial anteroposterior diameter (≥55 mm), were excluded. Those with significant coronary artery stenosis, organic heart disease, or systemic diseases were also excluded. Clinical data, echocardiographic findings, and imaging results were collected and recorded. This study was approved by the Ethics Committee of Taizhou Hospital and adhered to the principles of the Declaration of Helsinki.

### 2.2 Definition and classification of persistent atrial fibrillation

According to the 2017 Heart Rhythm Society expert consensus on catheter and surgical ablation of atrial fibrillation and the 2024 European Society of Cardiology guidelines for AF diagnosis and management, 1) Persistent AF: Defined as AF lasting continuously for more than seven days, including episodes terminated after seven days via pharmacological or electrical cardioversion. 2) Longstanding Persistent Atrial Fibrillation (LSPAF): Defined as AF lasting > 12 months. 3) Early Persistent Atrial Fibrillation (EPAF): Defined as AF lasting more than seven days but less than three months. Vagal Reflexes: Defined as intraoperative events, such as cardiac arrest, severe bradycardia, atrioventricular block, or hemodynamic changes, such as hypotension or shock due to the vagal response.

### 2.3 Study procedure

#### 2.3.1 Marshall vein chemical ablation

**Figure 2:**
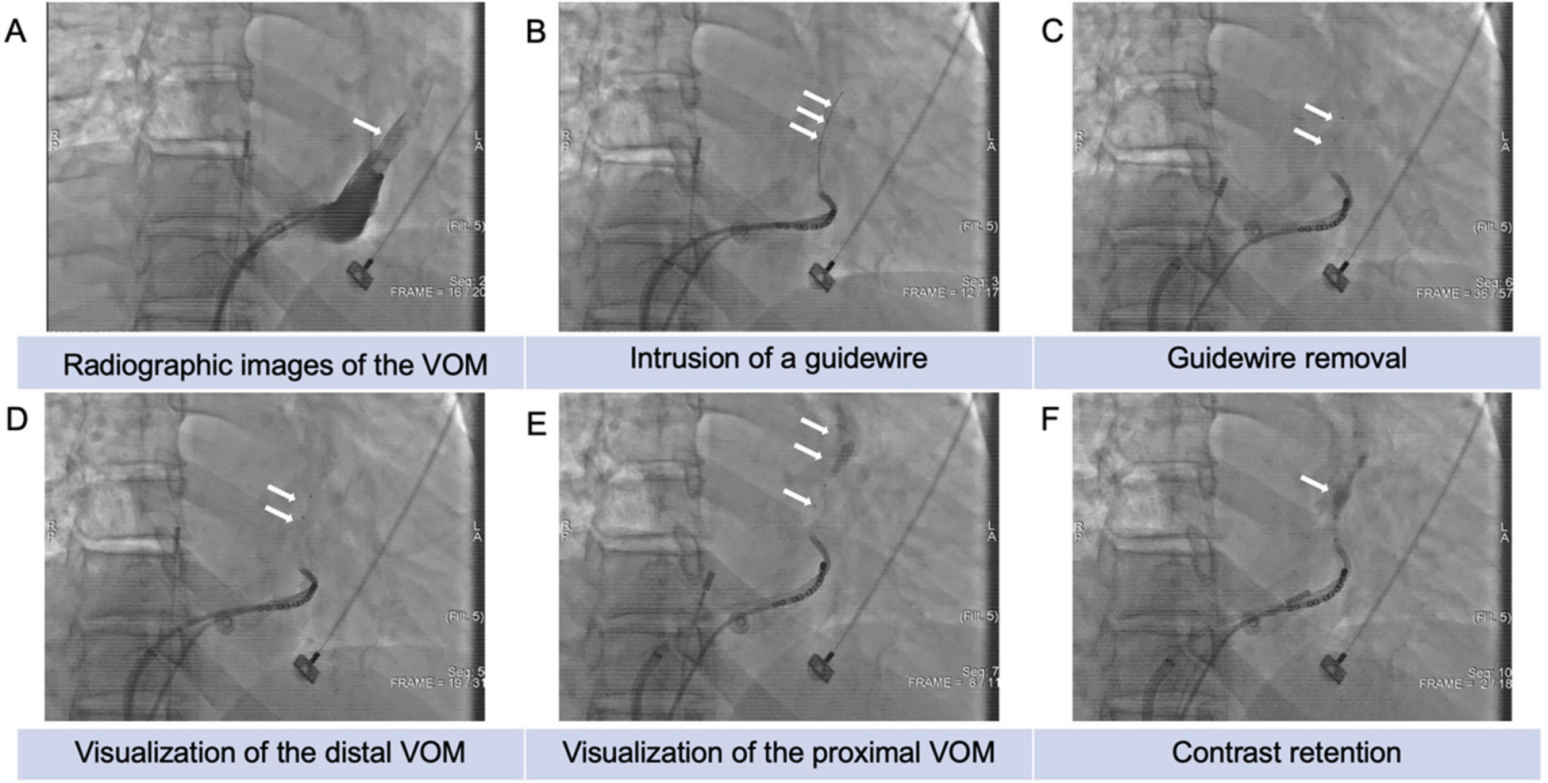
Steps of alcohol ablation of the vein of Marshall (VOM) under x-ray and venography **(A)** venography visualizing the VOM (white arrow). **(B)** Intrusion of a guidewire within the VOM (white arrow). **(C)** Inflation of the OTW balloon in the VOM to 6–8 atm, followed by guidewire removal. **(D)** Injection of contrast agent through the OTW balloon to visualize the distal VOM (white arrow). **(E)** Injection of contrast agent through the OTW balloon to visualize the proximal VOM (white arrow). **(F)** Contrast retention in the VOM region after ethanol injection (white arrow).

First, an 8.5F SL1 long sheath or steerable sheath was advanced to the coronary sinus (CS) ostium. Under a 30° right anterior oblique (RAO) fluoroscopic view, the SL1 or steerable sheath was used to introduce a 6F guiding catheter (JR4.0, Medtronic, MN) into the CS, directing the catheter toward the posterior-superior aspect of the CS. Contrast medium was injected incrementally, either distally or proximally, to locate the ostium of the VOM and perform selective venography of the Marshall vein. To obtain clearer imaging of the VOM and avoid overlapping with the CS, the RAO view was maintained throughout the procedure. If necessary, a mapping electrode was placed in the left pulmonary vein or the left atrial appendage as a reference. The JR4.0 catheter clockwise was rotated clockwise, and small amounts of contrast were injected at various positions to identify the VOM ostium. Next, a guidewire and an over-the-wire (OTW) balloon catheter (0.36 mm diameter BMW, 190 cm length; Abbott) were inserted through the JR4.0 catheter into the VOM. An appropriately sized balloon was selected based on the VOM diameter (1.5–2.5 mm in diameter, 8–12 mm in length; Boston Scientific). Alcohol ablation was performed in two stages: first, the OTW balloon was positioned at the distal VOM; second, it was retracted to the VOM ostium. The balloon was inflated to 6–8 atm at each site, and the guidewire was removed. Next, 1 mL of contrast medium was slowly injected through the balloon to obtain selective venography of the VOM (1:1 mixture of saline and contrast agent), ensuring complete occlusion without contrast reflux and assessment of the VOM anatomy and collateral circulation. Subsequently, over 1 min, 2 mL of 95% ethanol was slowly infused into the VOM, which was repeated twice at 1-minute intervals, totaling three infusions. The same procedure was applied to the proximal VOM with a maximum ethanol dose of 8–12 mL. After ethanol infusion, 1 mL of contrast medium (a 1:1 mixture of saline and contrast agent) was injected into the VOM to check for myocardial staining in the VOM region (Figure 2). Left atrial substrate mapping was performed before and after chemical ablation to evaluate the ablation area.

#### 2.3.2 Upgraded 2C3L ablation strategy

**Figure 3:**
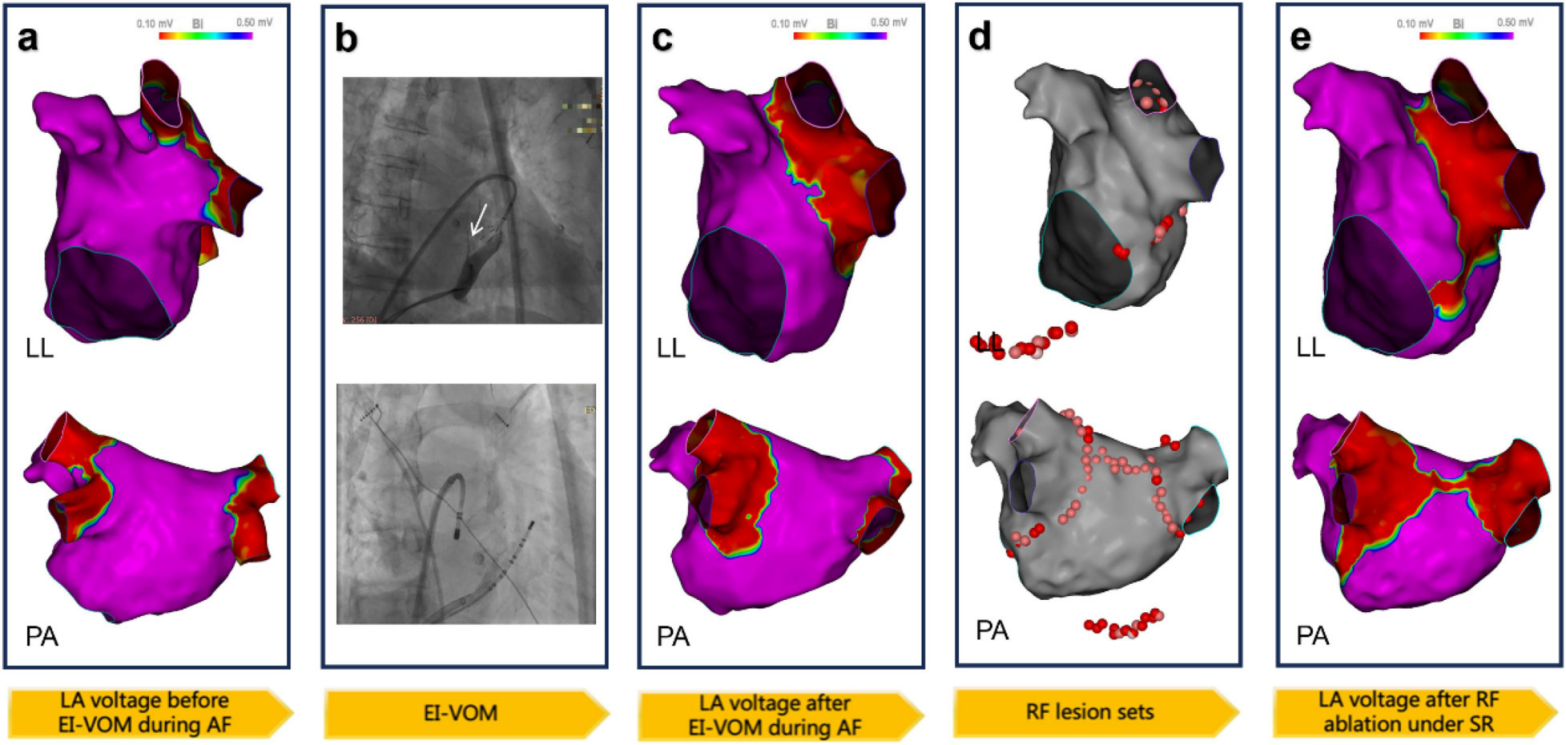
Procedure of the updated 2C3L ablation approach. **(a)** LA voltage mapping before EI-VOM during AF; **(b)** The upper panel shows the position and course of the Marshall vein (top panel, white arrow), and the lower panel shows contrast staining upon reinjection of the contrast agent through the OTW balloon after ethanol infusion (surrounded by a white dashed line); **(c)** After EI-VOM, voltage mapping revealed low-voltage areas in the mid-to-distal MI and the anterior and inferior margins of the left pulmonary veins, indicative of tissue injury from chemical ablation; **(d)** Subsequent bilateral pulmonary vein isolation and linear ablation were performed; within the chemically induced low-voltage zones, ablation power was reduced or omitted in regions with diminished local potentials; **(e)** After EI-VOM, repeat voltage mapping during sinus rhythm confirmed complete isolation of both pulmonary veins. Additionally, contiguous low-voltage areas (<0.1 mV) were observed at the left atrial roof and MI.

**Figure 4:**
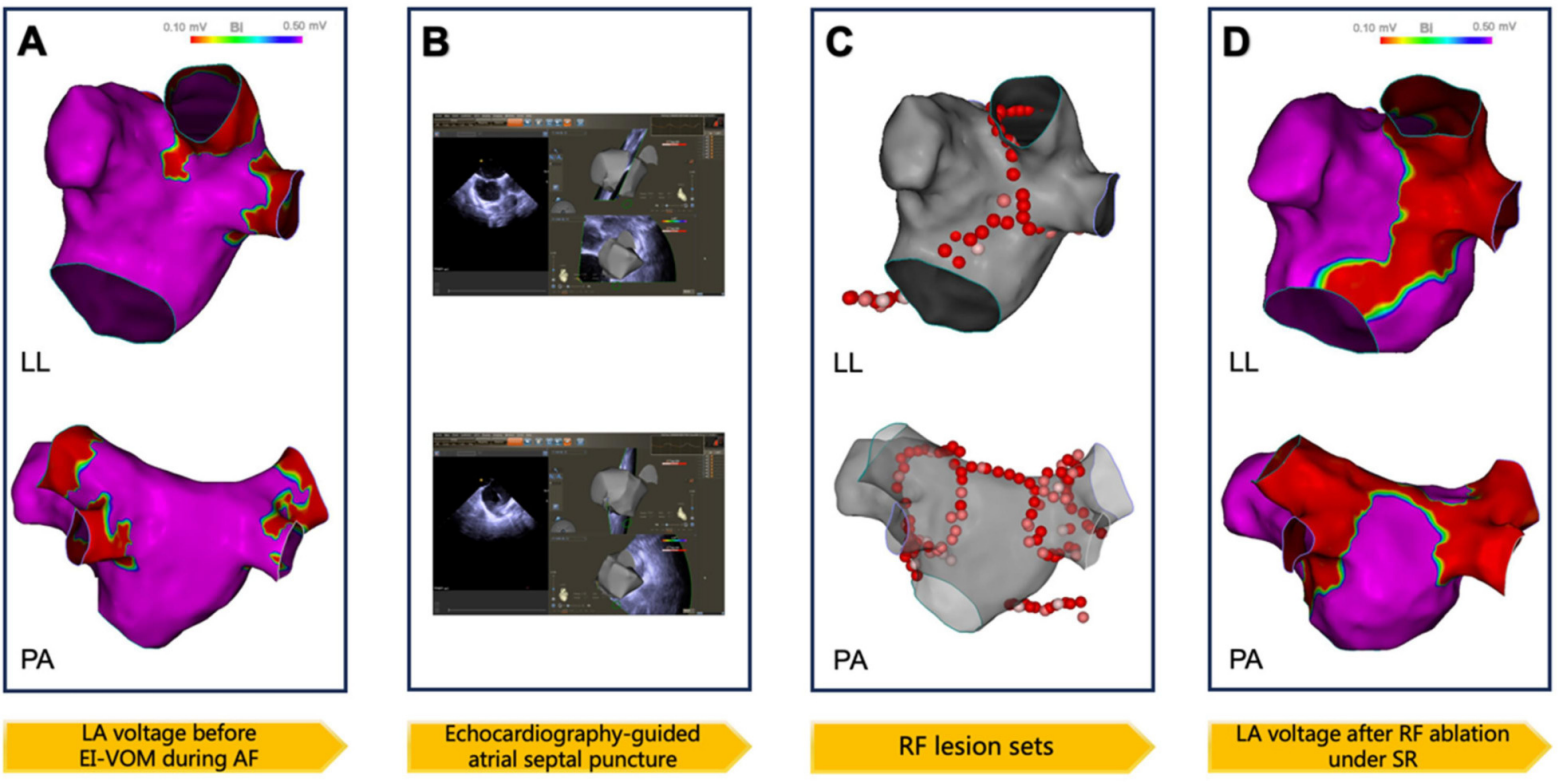
Procedure details of the ablation approach using RFCA alone. **(A)** LA voltage mapping before ablation during AF; **(B)** Schematic diagram of the x-ray combined with ICE-guided atrial septal puncture process; **(C)** Lesion sets of ablation LA voltage; **(D)** LA voltage under sinus rhythm after bilateral pulmonary vein isolation and linear ablation.

Under anesthesia, bilateral femoral venous access was established, and transseptal puncture was performed. A multi-electrode mapping catheter (PENTARAY) was positioned in the left atrium for substrate mapping. Subsequently, EI-VOM was performed, followed by substrate mapping. The detailed procedure of the updated 2C3L ablation approach in the EI-VOM + RFCA group is shown in Figure 3. Bilateral pulmonary vein isolation and flexion ablation were performed. During the procedure, 200 J synchronized electrical cardioversion was applied to restore sinus rhythm and confirm bilateral pulmonary vein isolation and roofline block. Furthermore, the ablation of the MI started from the annular side and extended along the isthmus to the low-voltage area induced by EI-VOM. Ablation was performed at the corresponding epicardial sites and the distal coronary sinus/great cardiac vein junction to block conduction through the coronary sinus muscle sleeve. The tricuspid isthmus line extended from approximately the 6 o'clock position of the tricuspid annulus to the inferior vena cava. If EI-VOM eliminated local potentials and created low-voltage areas, radiofrequency ablation was not performed. The procedural endpoint was complete bilateral pulmonary vein isolation with full conduction block across the three ablation lines. For patients in the RFCA alone group, the same 2C3L ablation strategy was implemented without the EI-VOM procedure. The specific procedural steps of RFCA alone are illustrated in Figure 4.

Bidirectional block was confirmed for the mitral isthmus line by differential pacing showing a > 120 ms delay or activation pattern change; for the left atrial roof line by activation detour around the superior vena cava or right pulmonary veins with widely separated double potentials; and for the cavotricuspid isthmus line by a strictly ascending activation wavefront from the low lateral right atrium to the septum with widely spaced double potentials along the line.

#### 2.3.3 Validation of procedure endpoints

After restoring sinus rhythm, conduction block was assessed as follows: The pulmonary vein potentials associated with atrial electrical activity were undetectable under sinus rhythm or coronary sinus pacing, indicating a conduction block. Following pulmonary vein isolation, a minimum observation period of 30 min was necessary. If the pulmonary vein conduction spontaneously recovered, additional ablation was performed. Pacing was then performed at the left superior, left inferior, right superior, and right inferior pulmonary veins as well as at the interpulmonary vein carina. If the pacing failed to capture the atrium, an efferent conduction block was considered. These assessments were conducted following sinus rhythm restoration to ensure the efficacy of the ablation procedure.

### 2.4 Follow-up

All patients underwent follow-up visits at 3, 6, and 12 months post-ablation, with subsequent follow-ups at 24 and 36 months. Evaluations included electrocardiography, echocardiography, and blood tests. Telephone follow-ups documented AF-related symptoms, such as palpitations, chest tightness, fatigue, and general discomfort. AF recurrence was defined as any atrial tachyarrhythmia lasting >30 s after the blanking period. The blanking period was the first three months post-ablation, during which no arrhythmias were considered an AF recurrence. Long-term success was defined as the absence of AF recurrence.

### 2.5 Statistical analysis

Continuous variables are presented as mean ± standard deviation (SD), while categorical variables are expressed as counts and percentages. Kaplan–Meier survival analysis was used to plot survival curves depicting post-ablation survival trends. Univariate and multivariate Cox proportional hazards regression models analyzed the factors influencing AF recurrence after cryoablation. Parametric or non-parametric tests were used to analyze continuous variables, whereas Fisher's exact test or chi-square test was used for categorical variables. Statistical significance was defined as *P* < 0.05. Statistical analyses were performed using SPSS (version 20.0; SPSS Inc., Chicago, IL, USA), and statistical figures were created using GraphPad Prism version 5.0 (GraphPad Software, San Diego, CA, USA). No subgroup or interaction analyses were performed due to limited sample size. To minimize potential bias, we used multivariable Cox regression to adjust for prespecified confounders (age, sex, LA diameter, AF duration) and censored patients at last follow-up.

## 3 Results

### 3.1 Postoperative follow-up analysis

A total of 112 patients completed the follow-up with a median duration of 35.0 months (interquartile range: 3.0–45.25 months). The baseline data are presented in Supplementary Table S1. Six patients with persistent AF dropped out or were lost to follow-up after catheter ablation. Among the 112 patients, 34 (30.36%) experienced AF recurrence, with two cases (1.86%) of atrial flutter (AFL) and one case (0.89%) of paroxysmal atrial tachycardia (AT). In the EI-VOM + RFCA group (*n* = 71), the median follow-up was 32.0 months (interquartile range: 3.0–37.5 months). One patient with persistent AF dropped out of the study after ablation. Among these, 16 patients (22.54%) experienced AF recurrence, with two cases (2.82%) of AFL and no cases of paroxysmal AT.

In our parallel study evaluating acute procedural outcomes, the acute bidirectional mitral isthmus block rate was significantly higher in the EI-VOM + RFCA group (96%, 69/72) than in the RF group (76%, 35/46) (*P* < 0.01). When these data were cross-referenced with the long-term follow-up cohort, patients in the EI-VOM + RFCA group who achieved acute MI block had numerically lower recurrence rates than those with residual conduction, suggesting that successful acute MI blockade may contribute to the superior long-term rhythm outcomes observed in the EI-VOM + RFCA group.

In the RFCA group (*n* = 41), the median follow-up was 47.5 months (interquartile range: 3.0–60 months), and five patients with persistent AF dropped out after ablation. Among these, 18 patients (43.90%) experienced AF recurrence, with no cases of AFL and one case (2.44%) of paroxysmal AT. The sinus rhythm maintenance rate was significantly higher in the EI-VOM + RFCA group than in the RFCA group (74.65% vs. 53.66%, *P* = 0.018).

**Figure 5:**
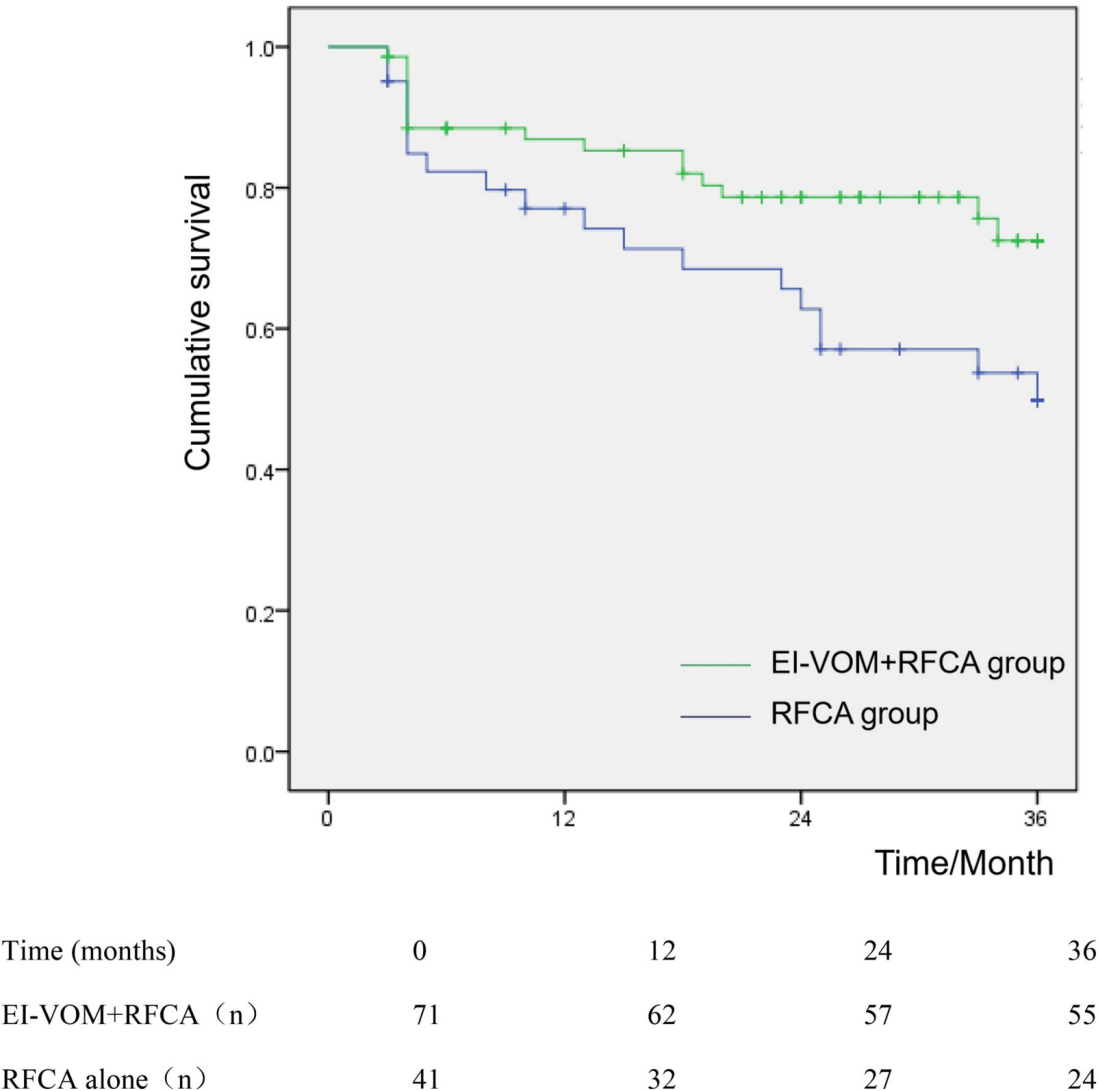
Sinus rhythm maintenance rate survival curve between the EI-VOM + RFCA and RFCA groups after catheter ablation.

The survival curve shows that non-AF survival at the follow-up terminus at 12 months among patients with PeAF was 83.93%, 75% at 24 months, and 70.54% at 36 months. The non-AF survival rate was 87.32% at 12 months in the EI-VOM + RFCA group, 80.25% at 24 months, and 77.46% at the 36-month follow-up point. The survival rate at 12 months was 78.05% in the RFCA group, 65.85% at 24 months, and 58.54% at the 36-month follow-up (*P* = 0.030, Figure 5). Patients who dropped out were censored at the time of last follow-up.

In our parallel study evaluating acute procedural outcomes, the acute bidirectional block rate for the mitral isthmus line was significantly higher in the EI-VOM + RFCA group than in the RFCA alone group (96% [69/72] vs. 76% [35/46], *P* < 0.01). For the left atrial roof line, the block rate in the EI-VOM + RFCA group was 94% (68/72) compared with 97.8% (45/46) in the RFCA alone group, indicating no superiority of EI-VOM for this line. For the cavo-tricuspid isthmus line, the block rate was 99% (71/72) in the EI-VOM + RFCA group vs. 93% (43/46) in the RFCA alone group (*P* = 0.11). Thus, the addition of EI-VOM significantly improved acute bidirectional block only for the mitral isthmus line, while its benefit for the roof line was not observed and for the cavo-tricuspid isthmus line was not statistically significant.

### 3.2 Procedural complications

No major complications, including cardiac tamponade, stroke, myocardial infarction, or death, occurred in either group. Minor complications were limited to three patients who developed small pericardial effusions that did not require intervention. Of these, two occurred in the EI-VOM + RFCA group and one in the RFCA alone group.

### 3.3 Analysis of AF recurrence post-ablation

Cox regression analysis identified a significant association between left atrial diameter (*P* = 0.06), EI-VOM (*P* = 0.043), and sinus rhythm maintenance rate after catheter ablation (Table 1). EI-VOM may help reduce the risk of AF recurrence by altering the electrophysiological properties of cardiac tissue. The Exp(B) value of 0.47 (0.23–0.98) indicated that patients undergoing alcohol ablation had an approximately 53% lower risk of AF recurrence compared to those who did not undergo this procedure. These findings suggest that EI-VOM may be an effective therapeutic strategy for improving sinus rhythm maintenance following radiofrequency ablation. As an adjunctive approach, alcohol ablation may reduce atrial electrical activity, improve AF recurrence rates, and improve patient prognosis. Although it carries a risk of complications, alcohol ablation may be a viable treatment option for carefully selected patient populations. An increase in left atrial diameter is associated with a higher risk of AF recurrence. The HR of 1.10 (1.03–1.18) indicates that each unit increase in left atrial diameter corresponded to a 10.1% increase in the risk of AF recurrence. Left atrial enlargement often reflects the persistence and chronicity of AF and may be related to atrial electrical remodeling. As an important risk factor for AF, an enlarged left atrium warrants greater attention in postoperative management. Enhanced pharmacological therapy or more aggressive interventions may be necessary to reduce recurrence risk in patients with larger LA diameters. Missing data for baseline variables were minimal (<5%) and handled by complete-case analysis. Sensitivity analyses were not performed due to the exploratory nature of this study.

**Table 1:** Factors influencing the recurrence of atrial fibrillation: (A) Sex; (B) Age; (C) duration of atrial fibrillation; (D) LA diameter; (E) hypertension; (F) diabetes mellitus; (G) smoking; (H) alcohol consumption.

Variables	B	SE	Wald	P	Exp(B)	Exp(B)的95% CI	
						Upper limit	Inferior limit
EIVOM	−0.758	0.375	4.081	0.043^*^	0.469	0.225	0.978
LA diameter	0.096	0.035	7.695	0.006^**^	1.101	1.029	1.179
BNP	0.000	0.001	0.001	0.976	1.000	0.999	1.001
LVEF	0.036	0.028	1.628	0.202	1.037	0.981	1.095
Sex	0.819	0.527	2.418	0.12	2.268	0.808	6.364
Age	−0.012	0.028	0.180	0.672	0.988	0.934	1.045
Cigarette	0.009	0.414	0.000	0.983	1.009	0.448	2.271
LDL	0.021	0.239	0.008	0.929	1.021	0.640	1.630
eGFR	0.004	0.017	0.043	0.836	1.004	0.971	1.038

* *P* < 0.05,

** *P* < 0.01.

## 4 Discussion

Our study offers two pivotal insights: (1) EI-VOM is associated with better sinus rhythm maintenance compared to the conventional 2C3L RFCA approach in patients with persistent AF; (2) this alternative strategy achieves higher rates of freedom from atrial arrhythmia over a 1–3 year follow-up period, making this study the first to report long-term outcomes of the EI-VOM combined with PVI line strategy.

AF recurrence after ablation is influenced by a complex interplay between mechanisms. PVI has been widely established as the cornerstone of AF ablation. However, PVI has limited effectiveness for persistent AF (13). To address this limitation, additional linear ablation strategies originating from the Cox maze surgical procedure were introduced to compartmentalize the atria and prevent AF maintenance (14). Nevertheless, these strategies have shown limited benefits in randomized trials (15, 16). This is mainly due to the intricate anatomy of the MI, where the great cardiac vein and VOM form a complex epicardial muscular network that precludes transmural lesions, making permanent MI block a great challenge (17, 18). Anatomically, VOM is an epicardial structure. Ethanol infusion achieves ablative effects through transmyocardial circulation, creating an epicardial-to-endocardial gradient that extends into the left atrial cavity (19). This approach enables rapid ablation of the atrial tissue near the VOM, particularly targeting regions commonly implicated in persistent AF. These include the lateral ridge of the left atrium, areas surrounding the left pulmonary veins (PVs), and regions extending toward the mitral annulus. Such regions frequently contribute to perimetral reentry (20), underscoring the strategic value of the VOM as a focal point during AF ablation procedures because of its distinct anatomical and functional characteristics (21).

The effectiveness of EI-VOM has been demonstrated in previous studies. The VENUS trial demonstrated that incorporating EI-VOM into catheter ablation significantly increased the probability of maintaining freedom from AF or AT at 6 and 12 months in patients with persistent AF (11). Similarly, the Marshall Plan, a prospective single-center study, reported a 79% freedom rate from recurrent AF/AT without the use of antiarrhythmic drugs, which was substantially higher than previously documented rates of 35%–70% (22). Furthermore, the recent randomized PROMPT-AF trial highlighted the value of EI-VOM in enhancing the 12-month outcomes of persistent AF ablation (12). Our preliminary findings also demonstrated that EI-VOM more effectively facilitates the MI conduction block (23). Earlier studies focused primarily on the feasibility and safety of EI-VOM. This study found that adding EI-VOM resulted in superior outcomes compared with RFCA alone. As the risk of arrhythmia recurrence increases over time, long-term follow-up is critical for assessing the durability of novel ablation strategies (24). Our findings indicated an arrhythmia-free survival rate of 87.32% at one year in the EI-VOM group compared to 78.05% in the RFCA group, 80.25% at two years in the EI-VOM group vs. 65.85% in the RFCA group, and 77.46% at three years in the EI-VOM group vs. 58.54% in the RFCA group. These results demonstrate the sustainable benefit of EIVOM beyond the 1-year follow-up period.

This study makes three key contributions to the field of PeAF ablation. First, the integration of EI-VOM with RFCA significantly improved long-term arrhythmia-free survival compared with RFCA alone, with a 77.46% vs. 58.54% success rate at 36 months. These findings align with previous short-term studies but extend their implications by demonstrating sustained efficacy, likely attributable to the dual mechanism of EI-VOM: (1) chemical denervation of VOM-associated autonomic ganglia, reducing trigger activity, and (2) creation of contiguous low-voltage zones that augment MI block durability (25). Second, left atrial diameter emerged as an independent predictor of recurrence, with each 1-mm increase increasing the recurrence risk by 10.1%. This underscores the role of structural remodeling in PeAF persistence, as atrial dilation fosters fibrosis and electrical heterogeneity, thereby perpetuating reentrant circuits (26). Third, our study also investigated the underlying mechanisms and examined individual factors that may influence the clinical outcomes and recurrence of atrial fibrillation (AF). Our results highlight the benefit of EI-VOM in patients with smaller left atria, suggesting that early intervention before advanced remodeling may optimize outcomes. However, the anteroposterior diameter of LA may not accurately reflect its true size (12). The left atrial volume (LAV) is a more reliable metric for assessing LA size, and patients with AF recurrence after RFCA exhibit significantly higher LA diameter. Considering the role of LAV in predicting prognosis following EI-VOM is essential (27).

The superiority of the EI-VOM aligns with anatomical insights. The proximity of VOM to the MI and pulmonary veins enables ethanol to target regions that are resistant to conventional RF energy, such as the epicardial muscle sleeves and Marshall bundles (18). Unlike RFCA, which often fails to achieve transmural lesions in the thick myocardium, retrograde diffusion of EI-VOM through the venous network of the VOM ensures broader tissue penetration (28). This mechanistic advantage was reflected in our higher MI block rates and reduced late PV reconnection, which is consistent with the finding of Nakashima et al. (29).

Nevertheless, our trial has several limitations, and the reasons for unsuccessful atrial arrhythmia termination or incomplete MI block remain unclear. Thomas et al. reported that even after EI-VOM, the protected epicardial musculature may maintain conduction across the MI, and electrical gaps are still observed in approximately one-third of repeat ablation procedures (30). Furthermore, some patients in our cohort who experienced arrhythmia recurrence underwent repeat procedures; however, documentation of lesion sites, including the identification and distribution of electrical gaps, was insufficient. Incorporating these pro-arrhythmic and anti-arrhythmic factors into the investigation of the effects of EI-VOM would provide valuable insights (31).Our findings may not be generalizable to centers without experience in EI-VOM or to patient populations with longer AF duration or more advanced left atrial remodeling.

## 5 Limitations

Despite these advances, the limitations warrant further investigation. First, procedural challenges, such as VOM stenosis or incomplete ethanol distribution, may explain the residual conduction in some patients. Thomas et al. reported persistent gaps in 30% of redo procedures post-EI-VOM (30), emphasizing the need for meticulous venography and staged ethanol delivery. Furthermore, it is important to note that only patients in whom EI-VOM was successfully performed were included in the EI-VOM + RFCA group; those in whom the procedure failed were excluded from the final analysis. Although this approach allows for an assessment of efficacy under optimal technical conditions, it may lead to an overestimation of the treatment effect and does not reflect the real-world applicability of the technique, including failure rates and underlying causes. To provide a more comprehensive perspective. Furthermore, due to the retrospective design, systematic left atrial voltage mapping was not performed in all patients, and low-voltage area data were not available for analysis. Additionally, left atrial volume index (LAVi) could not be calculated because volume measurements were not routinely reported during the study period; therefore, we used the anteroposterior diameter as a surrogate, which is a recognized limitation.Third, the lack of comprehensive follow-up data limits the interpretation of the subgroup analyses. Although we endeavored to enroll as many patients as possible, the power to assess the effectiveness of EI-VOM within each subgroup remained insufficient. Furthermore, the EI-VOM + RFCA group had a significantly higher proportion of patients with a shorter AF duration (<3 years), a known positive prognostic factor. Although we attempted to adjust for this in our multivariate model, the potential for residual confounding and selection bias inherent to the retrospective, non-randomized design remains a major limitation. Therefore, our findings should be considered hypothesis-generating, and prospective randomized trials with balanced follow-up are required to confirm the long-term superiority of EI-VOM. Further validation through larger-scale studies with a more balanced representation of sexes, involvement of multiple operators from different centers, and the use of a randomized design is essential. Future studies incorporating continuous monitoring strategies are needed to more accurately assess the true arrhythmia burden and recurrence rates. Such studies offer deeper insight into the efficacy of the EI-VOM strategy and its potential to improve long-term outcomes in patients with persistent AF. We anticipate that these investigations will contribute to the establishment of EI-VOM as a standard adjunctive approach for the treatment of persistent AF.

## 6 Conclusion

In summary, our study demonstrated better sinus rhythm maintenance in patients undergoing EI-VOM than in those undergoing conventional RFCA in the management of persistent AF. EI-VOM represents a paradigm shift in PeAF ablation that addresses the anatomical and electrophysiological limitations of conventional RFCA. By targeting the unique substrate of the VOM, this strategy enhances long-term rhythm control, particularly in patients with less advanced atrial remodeling. Future research should focus on optimizing ethanol delivery protocols and exploring synergistic approaches such as hybrid ablation to further improve outcomes.

## Data Availability

The original contributions presented in the study are included in the article/Supplementary Material, further inquiries can be directed to the corresponding authors.
