# Supplementary material for: Long-term clinical efficacy of Marshall vein ethanol infusion combined with catheter ablation for persistent AF
Source: Front Cardiovasc Med. 2026 May 12;13:1809308. doi: 10.3389/fcvm.2026.1809308 (PMC13203990; doi:10.3389/fcvm.2026.1809308)
Supplement: Supplementary file 1 [file Datasheet1.docx]

**Supplement Table 1. Baseline characteristics**

| **Characteristic** | **RF（n=46）(Number (%) or mean±SD)** | **VOM-Et-RF（n=72）(Number (%) or mean±SD)** | **P value for *X*^2^-test or t-test** |
| --- | --- | --- | --- |
| Age (y) | 62.67±8.7 | 64.31±8.3 | 0.663 |
| Male sex | 30(65.2) | 48(66.6) | 0.513 |
| **Medical history and Risk factors** |  |  |  |
| Hypertension | 25 (54.3) | 63(87.5) | 0.705 |
| Diabetes | 4(8.7) | 8(11.1) | 0.464 |
| Coronary disease | 1(2.2) | 2(2.8) | 0.664 |
| Stroke/TIA | 0(0) | 0(0) | - |
| Heart failure | 0(0) | 0(0) | - |
| Body mass index (kg/m2)* | 24.93±2.8 | 25.13±3.4 | 0.054 |
| CHA2DS2-VASc score† | 1.48±1.07 | 1.65±1.26 | 0.424 |
| **Cardiac parameters** |  |  |  |
| Ejection fraction (%) | 58.52±8.5 | 56.92±8.0 | 0.643 |
| Left atrial diameter (mm) | 43.61±5.6 | 43.64±5.2 | 0.805 |
| **Time from first AF diagnosis** |  |  | 0.344 |
| <3 y | 23 | 43 |  |
| >3 y | 23 | 29 |  |


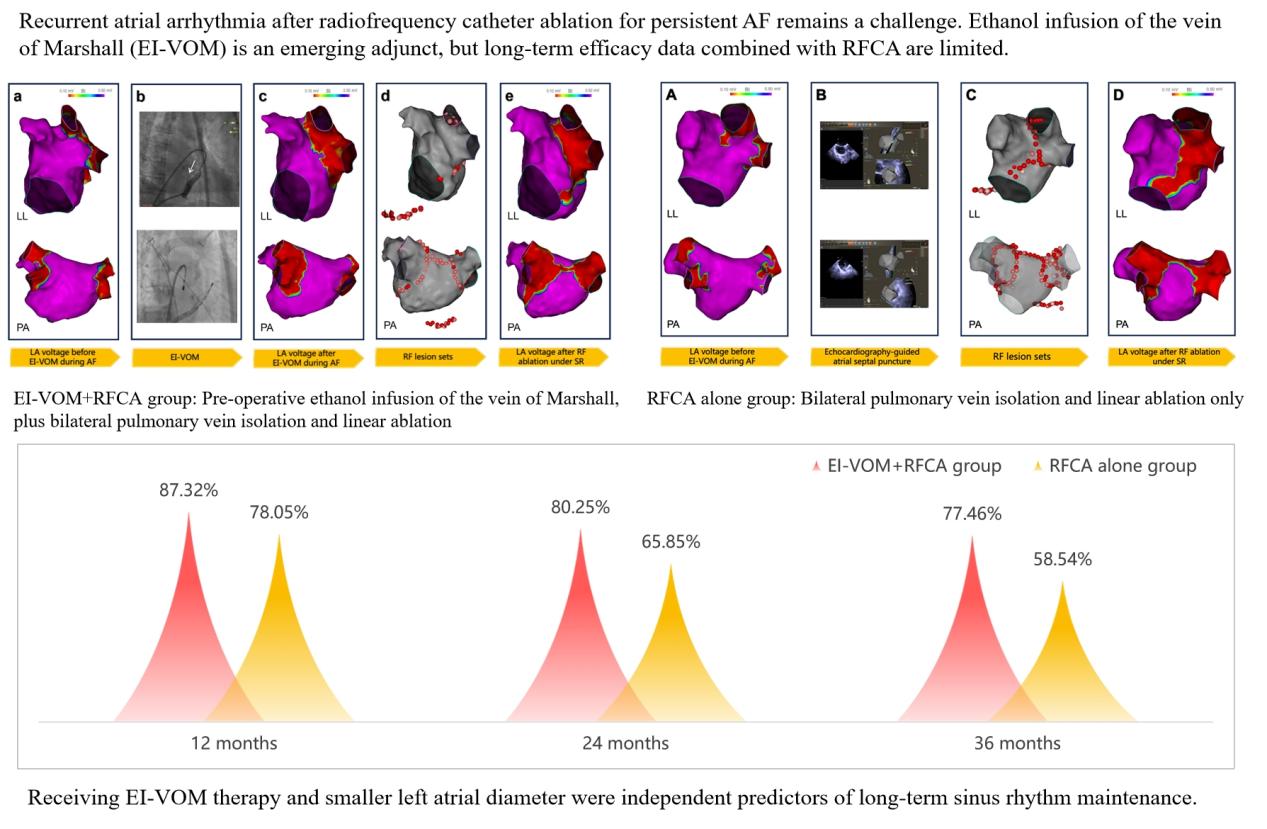


**Supplement Figure 1. A Visual abstract**
